# Premio Nobel de Fisiología o Medicina 2026: cómo, a partir de la curiosidad, se construye conocimiento importante para la vida.

**DOI:** 10.7705/biomedica.8711

**Published:** 2026-10-16

**Authors:** Orlando Torres-Fernández

**Affiliations:** 1 Grupo de Morfología Celular, Dirección de Investigación en Salud Pública, Instituto Nacional de Salud, Bogotá, D. C., Colombia Grupo de Morfología Celular Dirección de Investigación en Salud Pública Instituto Nacional de Salud Bogotá, D. C. Colombia

La investigación básica se define como aquella que se lleva a cabo sin saber si el conocimiento que se pueda adquirir se traducirá a corto, mediano o largo plazo, en alguna utilidad práctica. Es la investigación motivada por la curiosidad. A partir de la observación de hechos que ocurren espontáneamente en la naturaleza o que son detectados en experimentos de laboratorio, el investigador desea encontrar una explicación de lo que está observando y aplica el método científico para lograrlo. De los primeros resultados obtenidos surgen nuevas preguntas para responder y se inicia, así, una cadena de sucesos que da lugar a una línea de investigación a la cual pueden también unirse otros investigadores en la medida que se den a conocer los resultados publicados.

El Premio Nobel de Medicina o Fisiología 2026 es un ejemplo de cómo la investigación básica ha sido un pilar del desarrollo del conocimiento que, finalmente, conduce a facilitar el análisis y a encontrar soluciones a los retos a los que debe enfrentarse la humanidad. Este año el reconocimiento fue otorgado a los investigadores Karl Deisseroth, Peter Hegemann y Georg Nagel «Por sus descubrimientos sobre los canales iónicos regulados por la luz y la optogenética».

Desde mediados del siglo XIX, existen registros del fototropismo de algunas algas unicelulares que se desplazan mediante el batido de sus flagelos. Un famoso botánico ruso de la época, Andrei Faminzyn (1835-1918), llevó a cabo estudios al respecto en algas de los géneros *Chlamydomonas* y *Euglena*. Sin embargo, durante más de un siglo se desconoció el mecanismo que dirigía el movimiento de estas algas para acercarse o alejarse de la luz. Fue apenas en la década de los ochenta del siglo pasado que se encontró la respuesta, pero el esclarecimiento del mecanismo estuvo precedido de importantes hallazgos obtenidos en microorganismos muy primitivos no fotosintéticos (Haloarquea).

Las arqueas son similares morfológicamente a las bacterias y pueden encontrarse en ambientes con características extremas. La especie *Halobacterium salinarum* (anteriormente *H. halobium*) se encuentra en ambientes con altas concentraciones de sal y es fácil de cultivar. En 1971, se descubrió la composición química de una parte de su membrana celular que contiene un pigmento púrpura fotosensible, es decir que reacciona a la luz, con el propósito de activar una bomba para extraer protones hacia el espacio exterior y por su composición química similar a la de las rodopsinas de los bastones de los vertebrados, se le llamó bacteriorrodopsina ^1^. Poco después, en este mismo microorganismo se descubrió otro pigmento similar, pero con función diferente: la incorporación de iones cloruro desde el espacio extracelular. A este se le llamó halorrodopsina.

Peter Hegemann se interesó en el tema desde que inició sus estudios universitarios de pregrado en química en la década de los años setenta y, en 1984, presentó su tesis doctoral titulada «La halorrodopsina, la bomba de cloruro activada con luz en *Halobacterium halobium*» con la que profundizó en el conocimiento de su estructura y función. Paralelamente, Foster, *et al*. ^2^ descubrieron que una rodopsina era el fotorreceptor determinante del fototropismo de las algas del género *Chlamydomonas* (un siglo después de las observaciones de Faminzyn). Encontraron que la composición química era muy similar a la ya conocida de las rodopsinas microbianas (bacteriorrodopsina y halorrodopsina). Esto llamó la atención de Hegemann, quien decidió realizar una estancia postdoctoral con el grupo de Foster. Posteriormente, como investigador del instituto Max Planck, Hegemann continúo dedicado a profundizar en el conocimiento de las rodopsinas del alga unicelular *Chlamydomonas reinhardtii*; hasta ahora, continúa siendo su principal tema de investigación, con más de cien artículos publicados.

Por otra parte, el biólogo Georg Nagel, también investigador de la prestigiosa institución alemana, fue el pionero en «inyectar» el gen de la bacteriorrodopsina en ovocitos del anfibio *Xenopus leavis.* La expresión del gen los hizo sensibles a la luz para activar una bomba de protones hacia el espacio extracelular ^3^. Este experimento podría considerarse el origen de la optogenética, aunque el término fue acuñado años después cuando se hicieron los primeros estudios para introducir los genes de las rodopsinas en neuronas.

Empezando el siglo XXI, Nagel y Hegemann unieron esfuerzos. Un investigador del equipo de trabajo de Hegemann había encontrado recientemente en una genoteca dos nuevas secuencias de ADN de rodopsinas de tipo microbiano en *C. reinhardtii*. Nagel logró expresar las dos rodopsinas en los ovocitos de *Xenopus*. Sorprendentemente, los dos ADN codificaban para canales catiónicos activados directamente por la luz. Fue así como descubrieron una clase nueva de rodopsinas, las rodopsinas de canal: canalrodopsina-1 (ChR1) ^4^ y canalrodopsina-2 (ChR2) ^5^.

Los investigadores también lograron expresar ChR2 en células embrionarias de riñón humano (HEK-293) y en fibroblastos derivados de riñón de hámster (BHK) ^5^, abriendo así un gran campo de investigación sobre la despolarización de membranas inducida por la luz. Nagel y Hagemann motivaron el interés de diferentes investigadores en el mundo a quienes suministraron los genes, especialmente el ChR2, de tal manera que, para el año 2006, ya se había demostrado la funcionalidad de esta canalrodopsina en la retina de ratones ciegos, en neuronas del hipocampo, en la médula espinal de embriones de pollos vivos y en rodajas de cerebro de ratón, entre otros sustratos. Diez años después, los investigadores escribieron: «Cuando concebimos y realizamos nuestros experimentos hace una década, no esperábamos que la investigación sobre el mecanismo molecular de la fototaxis de las algas o el transporte iónico impulsado por la luz en las arqueas pudiera interesar a los lectores de una revista médica» ^6^.

Karl Deisseroth, quien para la época del descubrimiento de las canalrodopsinas era un joven neurocientífico estadounidense, fue quien mejor aprovechó los avances de los investigadores alemanes. El investigador utilizó el canal catiónico fotosensible ChR2 como herramienta para el control temporal, preciso y no invasivo de la actividad en poblaciones neuronales, de tal manera que se pudiera utilizar la luz para generar potenciales de acción individual y otros eventos sinápticos, así como descifrar la conectividad en redes neuronales. Inicialmente, introdujo la canalrodopsina en neuronas individuales y, luego, desarrolló el método para hacerlo en el cerebro de ratones ^7^^-^^9^. Deisseroth *et al*. utilizaron por primera vez el término «optogenética» para referirse a la disciplina que «combina la selección genética de neuronas o proteínas específicas con la tecnología óptica para la obtención de imágenes o el control de los objetivos dentro de circuitos neuronales vivos e intactos». En otras palabras, «con el advenimiento de los métodos ópticos y genéticos combinados se pueden visualizar neuronas modificadas genéticamente en animales vivos, así como rastrear y controlar eventos eléctricos y bioquímicos dentro de los tipos celulares seleccionados» ^9^.

Para el 2011, la optogenética había evolucionado hasta desarrollar modificaciones a las canalrodopsinas y descubrir o crear nuevas de ellas, así como realizar ajustes a las metodologías para adaptarlas a diferentes necesidades específicas de la investigación en neurociencias. Karl Deisseroth fue protagonista de muchos de esos avances ^10^. A esto se suma el hecho de que su grupo fue el creador del método CLARITY, un procedimiento que permite hacer transparente el tejido nervioso en ratones eliminando los lípidos de la membrana celular, pero conservando intactas y fácilmente visibles las redes y conexiones neuronales ^11^. Deisseroth es un médico psiquiatra, pero, como él mismo lo reconoce, su formación inicial en bioquímica le facilitó incursionar en el campo de investigación de la neurociencia básica. Y, si bien la optogénetica aún no se ha desarrollado a un nivel terapéutico, sí ha contribuido al conocimiento de la etiología de muchas enfermedades neurológicas. Deisseroth, empleando la optogenética, ha contribuido en el estudio de algunas de ellas ^12^^,^^13^.

Aquí vale la pena citar a Francis Crick (premio nobel 1962), quien había resaltado la dificultad de usar electrodos para distinguir entre diferentes tipos de neuronas en los cerebros de mamíferos, destacando que era necesario encontrar un método para controlar con precisión la actividad de un tipo neuronal sin afectar los demás y, especulando, sugirió que la luz podría ser la herramienta ^14^, aunque sin formular hipótesis alguna. No obstante, se le podría llamar el «profeta de la optogenética».

Este premio nobel reconoció el trabajo de los investigadores que realizaron diferentes aportes a la investigación básica de una misma temática que se construyó durante décadas, estudiando un mismo tipo de moléculas en organismos tan diferentes como las arqueas y los eucariotas. Como ocurre con el otorgamiento de este y otros galardones, quedan por fuera de los reconocimientos muchos investigadores casi anónimos, como quien halló en una genoteca los primeros dos genes de las canalrodopsinas y otros que realizaron aportes tan importantes como el de haber identificado las opsinas microbianas (halorrodopsina y la bacteriorrodopsina) en las arqueas o los fotorreceptores en las algas unicelulares del género *Chlamydomonas.* Por mucho menos, las películas de Hollywood incluyen al final una extensa lista de colaboradores, por qué no hacer algo así cuando se otorga un premio nobel en ciencias en en el cual muchos investigadores han hecho su contribución.
